# The HSP90 inhibitor ganetespib has chemosensitizer and radiosensitizer activity in colorectal cancer

**DOI:** 10.1007/s10637-014-0095-4

**Published:** 2014-04-01

**Authors:** Suqin He, Donald L. Smith, Manuel Sequeira, Jim Sang, Richard C. Bates, David A. Proia

**Affiliations:** Synta Pharmaceuticals Corp, 125 Hartwell Avenue, Lexington, MA 02421 USA

**Keywords:** HSP90 inhibition, Ganetespib, Colorectal cancer, Combination therapy

## Abstract

The integration of targeted agents to standard cytotoxic regimens has improved outcomes for patients with colorectal cancer (CRC) over recent years; however this malignancy remains the second leading cause of cancer mortality in industrialized countries. Small molecule inhibitors of heat shock protein 90 (HSP90) are one of the most actively pursued classes of compounds for the development of new cancer therapies. Here we evaluated the activity of ganetespib, a second-generation HSP90 inhibitor, in models of CRC. Ganetespib reduced cell viability in a panel of CRC cell lines in vitro with low nanomolar potency. Mechanistically, drug treatment exerted concomitant effects on multiple oncogenic signaling pathways, cell cycle regulation, and DNA damage repair capacity to promote apoptosis. Combinations of ganetespib and low-dose ionizing radiation enhanced the radiosensitivity of HCT 116 cells and resulted in superior cytotoxic activity over either treatment alone. In vivo, the single-agent activity of ganetespib was relatively modest, suppressing HCT 116 xenograft tumor growth by approximately half. However, ganetespib significantly potentiated the antitumor efficacy of the 5-Fluorouracil (5-FU) prodrug capecitabine in HCT 116 xenografts, causing tumor regressions in a model that is intrinsically resistant to fluoropyrimidine therapy. This demonstration of combinatorial benefit afforded by an HSP90 inhibitor to a standard CRC adjuvant regimen provides an attractive new framework for the potential application of ganetespib as an investigational agent in this disease.

**Electronic supplementary material:**

The online version of this article (doi:10.1007/s10637-014-0095-4) contains supplementary material, which is available to authorized users.

## Introduction

In spite of welcome declines in the mortality rate over the past two decades, colorectal cancer (CRC) remains the second leading cause of cancer death among adults living in industrialized countries. In fact, 2013 estimates predict for more than 140,000 new cases and 50,000 deaths due to this disease in the United States alone [1]. Advances in, and greater use of, available screening techniques have resulted in earlier diagnoses with subsequent medical intervention and thus represent major contributing factors for the observed decrease in CRC-related mortality [2]. Further, the introduction of newer chemotherapeutic drugs and treatment regimens, including those that incorporate targeted agents, have led to meaningful improvements in the median overall survival time for patients with metastatic CRC [3]. Despite this progress however, the prognosis for individuals with unresectable advanced disease continues to be grave and there still exists a substantial unmet need for novel therapeutic approaches to improve clinical outcomes in this malignancy.

The molecular chaperone heat shock protein 90 (HSP90) regulates the maturation and functional stability of an extensive array of cellular target substrates, termed “client” proteins [4]. Beyond an essential role in maintaining normal tissue homeostasis, the chaperoning activity of HSP90 is now recognized as critical for the function of many of these same clients, as well as mutated and aberrantly expressed forms, which contribute to nearly every aspect of the tumorigenic process including immortality, survival, metabolism, angiogenic, and/or metastatic potential [5, 6]. Inhibiting HSP90 activity triggers the ubiquitination and proteasomal degradation of its client proteins, in turn providing a highly effective means to simultaneously disrupt multiple oncogenic signaling cascades through a singular molecular target [7, 8]. This unique characteristic distinguishes this therapeutic strategy from more traditional targeted approaches, such as kinase inhibition, that selectively ablate only one or a few oncoproteins. Pharmacological blockade of HSP90 has therefore emerged as an innovative and multifaceted approach for the development of new antineoplastic agents for a variety of human cancers [9, 10].

Ganetespib is an investigational small molecule inhibitor of HSP90 with favorable pharmacologic properties that distinguish the compound from other first- and second-generation HSP90 inhibitors in terms of potency, safety, and tolerability [11, 12]. Ganetespib has been shown to possess robust antitumor activity against a variety of cancer types in preclinical studies, including lung, breast, and prostate [13–18]. Moreover, the early clinical evaluation of ganetespib has revealed encouraging signs of single-agent therapeutic activity in human tumors. Most notably these have been observed in a molecularly defined subset of non-small cell lung cancers oncogenically dependent on EML4-ALK gene rearrangements [19], the fusion protein products of which are highly sensitive to ganetespib exposure [20]. Interestingly, as part of the initial Phase I study of ganetespib in patients with solid malignancies, the most significant demonstration of clinical efficacy involved a patient with metastatic CRC who achieved a partial response (PR) while on-therapy [21]. This provocative finding therefore prompted a more comprehensive evaluation of ganetespib activity in this malignancy. The results of the present study suggest that ganetespib may hold considerable promise, particularly as part of combinatorial-based strategies, for the treatment of advanced CRC.

## Materials and methods

### Cell lines, antibodies, and reagents

All colorectal cell lines with the exception of COLO-678 were obtained from the American Type Culture Collection (ATCC, Manassas, VA, USA) and maintained at 37 °C in 5 % (v/v) CO_2_ using culture medium recommended by the supplier. COLO-678 cells were obtained from DSMZ (German Collection of Microorganisms and Cell Cultures, Braunschweig, Germany). All primary antibodies were purchased from Cell Signaling Technology (CST, Beverly, MA, USA) with the exception of the GAPDH antibody (Santa Cruz Biotechnology Inc., Santa Cruz, CA). Ganetespib [3-(2,4-dihydroxy-5-isopropylphenyl)-4-(1-methyl-1H-1,2,4-triazol-5(4H)-1] was synthesized by Synta Pharmaceuticals Corp. 5-Fluorouracil and capecitabine were purchased from Sigma-Aldrich (St. Louis, MO, USA) and bevacizumab was obtained from the Dana Farber Cancer Institute (Boston, MA, USA).

### Cell viability assays

Cellular viability was assessed using the CellTiter-Glo Luminescent Cell Viability Assay (Promega, Madison, WI, USA) according to the manufacturer’s protocol. Colorectal cancer cell lines were seeded into 96-well plates based on optimal growth rates determined empirically for each line. Twenty-four hours after plating, cells were dosed with graded concentrations of drug for 72 h. CellTiter-Glo was added (50 % *v*/*v*) to the cells, and the plates incubated for 10 min prior to luminescent detection in a Victor 2 microplate reader (Perkin Elmer, Waltham, MA, USA). Data were normalized to percent of control and IC_50_ values were determined using XLFit software.

### Flow cytometry

For cell cycle analysis, HCT 116 cells were seeded overnight in a 6-well plate and then exposed to increasing concentrations of ganetespib (10–1,000 nM) or vehicle (DMSO) for 18 h. Cells were harvested and stained with propidium iodide using the BD Cycle TEST PLUS Reagent Kit (BD Biosciences, San Jose, CA, USA) according to the manufacturer’s instructions. Twenty thousand cells were analyzed for their DNA content using a FACS Caliber cytometer (BD Biosciences, Billerica, MA, USA). For the apoptosis assay, cells were treated over the same range of ganetespib concentrations for 24 h. Following treatment, cells were harvested and stained using a fluorescein-conjugated anti-Annexin V antibody (BD Biosciences) and apoptosis assessed by flow cytometry.

### Western blotting

Following in vitro assays, tumor cells were disrupted in lysis buffer (CST) on ice for 10 min. Lysates were clarified by centrifugation and equal amounts of proteins resolved by SDS-PAGE before transfer to nitrocellulose membranes (Bio-Rad, Hercules, CA, USA). Membranes were blocked with Starting Block T20 blocking buffer (Thermo Scientific, Cambridge, MA, USA) and immunoblotted with the indicated antibodies. Antibody-antigen complexes were visualized using an Odyssey system (LI-COR, Lincoln, NE, USA).

### Combination drug and irradiation treatment

Exponentially growing HCT 116 cell cultures were treated with increasing concentrations of ganetespib either alone or concurrent with exposure to ionizing radiation. Irradiation was performed at room temperature using a Cesium 137 Mark I Irradiator (JL Shepherd and Associates, San Fernando, CA, USA) to a final dose of 2 Gy. Cells were similarly treated in parallel with DMSO as vehicle controls. At 48 h post-irradiation, cells were harvested and subject to Annexin V analysis by flow cytometry. HCT 116 cells treated with graded concentrations of ganetespib with or without simultaneous ionizing radiation were additionally harvested at 24 h and protein expression changes evaluated by Western blot. For microscopic studies, cells were treated with 100 nM ganetespib or 2 Gy irradiation, either alone or in combination, for 48 h. Cells were washed, fixed, and permeabilized before staining with Alexa Fluor® 594 phalloidin (Life Technologies, Grand Island, NY, USA) for 30 min. Slides were mounted using DAPI-containing mounting medium (VECTASHIELD, Vector Labs, Burlingame, CA, USA) and images obtained using an EVOS-FL fluorescent microscope (Life Technologies).

### In vivo xenograft models

CD-1 nude mice (Charles River Laboratories, Wilmington, MA) at 7–12 weeks of age were maintained in a pathogen-free environment and all in vivo procedures were approved by the Synta Pharmaceuticals Corp. Institutional Animal Care and Use Committee. HCT 116 (5 × 10^6^) cells were subcutaneously implanted into female mice and animals bearing established tumors (~150 mm^3^) were randomized into treatment groups of 8. For evaluating single-agent activity, mice were dosed with vehicle or 150 mg/kg ganetespib (i.v.) formulated in DRD (10 % DMSO, 18 % Cremophor RH 40, 3.6% dextrose) on a weekly schedule. For the combination experiment, animals were treated over a 3 week cycle as follows: i.v. ganetespib (150 mg/kg) once a week, p.o. capecitabine (400 mg/kg) daily for the first 14 days, or both regimens in combination. Tumor volumes (V) were calculated by caliper measurements of the width (W), length (L) and thickness (T) of each tumor using the formula: *V* = 0.5236(LWT). Tumor growth inhibition was determined from the change in average tumor volumes of each treated group relative to the vehicle-treated, or itself in the case of tumor regression. Statistical significance was determined using two-way ANOVA followed by Bonferroni post tests.

## Results

### Inhibition of oncogenic signaling pathways by ganetespib induces cell death and suppresses tumor growth in colon cancer models

**Fig. 1:**
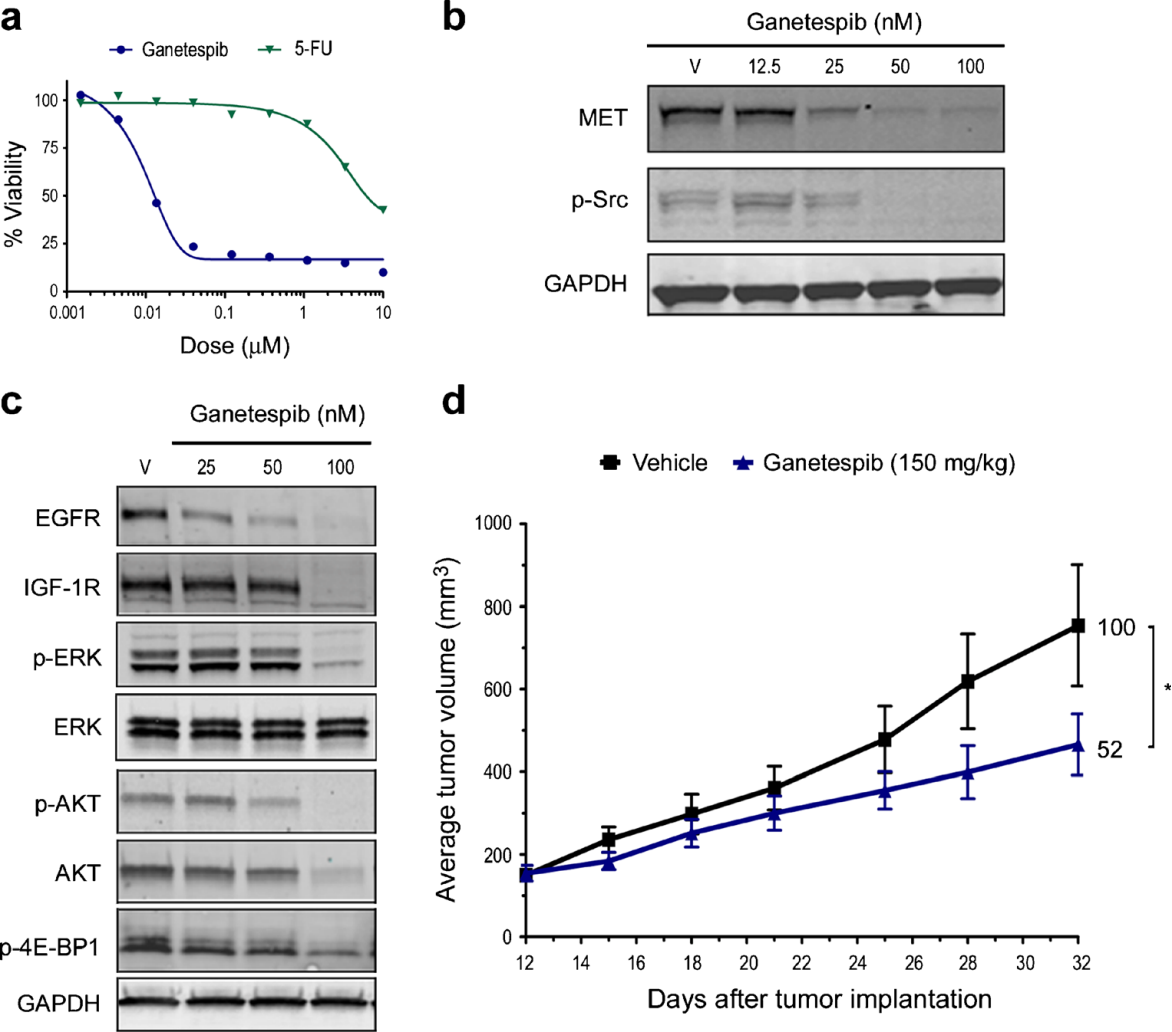
Ganetespib activity in HCT 116 colon cancer cells in vitro and in vivo. **a** HCT 116 cells were treated with increasing concentrations of ganetespib or 5-FU and cell viability assessed after 72 h. **b** HCT 116 cells were exposed to graded concentrations of ganetespib or vehicle (V) for 24 h as indicated. Cell lysates were immunoblotted using antibodies against MET and phosphorylated Src (p-Src) as shown. GAPDH was included as a loading control. **c** HCT 116 cells were exposed to vehicle or ganetespib (25, 50 and 100 nM) for 24 h as indicated. Cell lysates were immunoblotted using antibodies against EGFR, IGF-1R, phosphorylated ERK (p-ERK), total ERK, phosphorylated AKT (p-AKT), total AKT, and phosphorylated 4E-BP1 (p-4E-BP1). **d** Mice bearing established HCT-116 xenografts (*n* = 8/group) were i.v. dosed with 150 mg/kg ganetespib once weekly over a 3 week cycle. % T/C values are indicated to the right of each growth curve and the error bars are the SEM; (*, *p* < 0.05)

Initially, the cytotoxic activity of ganetespib was evaluated using a panel of 15 CRC lines, where it reduced cell viability with low nanomolar potency (Table 1). Of interest, the two most sensitive lines, RKO and LS-411 N, both harbor an activating BRAF^V600E^ mutation and we have recently reported that expression of this oncogenic driver and established HSP90 client confers sensitivity to ganetespib in BRAF^V600E^-driven melanoma cell lines [22]. One additional line in the panel, COLO-205, bears the same BRAF mutation and these cells were also highly sensitive to ganetespib exposure (IC_50_, 14 nM). Subsequently we investigated the effects of ganetespib exposure using the well-characterized HCT 116 cell line as our model system. Ganetespib potently reduced viability in HCT 116 cells with an IC_50_ value of 14 nM (Table 1, Fig. 1a). By comparison, the cells were largely insensitive to the cytotoxic effects of the standard-of-care chemotherapeutic 5-Fluorouracil (5-FU), which had an IC_50_ value of approximately 10 μM (Fig. 1a).

**Table 1:** In vitro cytotoxicity values of ganetespib in colorectal cancer lines

Cell line	Ganetespib IC_50_ (nM)
RKO	4
LS-411 N	5
SW620	8
HCT-15	8
HuTu-80	13
HCT 116	14
COLO-205	14
NCI-H747	17
COLO-678	21
LoVo	22
LS-1034	31
SNU-C2B	45
LS-123	73
SK-CO-1	81
HCC2998	128

Next we examined expression changes in HSP90 client proteins and signaling pathways associated with colon cancer progression. As shown in Fig. 1b, ganetespib treatment resulted in a dose-dependent destabilization of MET receptor tyrosine kinase expression in HCT 116 cells and this was accompanied by inactivation of one of its downstream effector pathways, as evidenced by the loss of phosphorylated Src activity. Ganetespib exposure also promoted the dose-dependent degradation of EGFR and IGF-1R receptors, loss of AKT signaling activity (shown by reductions in both total and phosphorylated AKT protein levels), and decreased expression of phosphorylated 4E-BP1, indicative of disruption of the mTOR (mammalian target of rapamycin) pathway (Fig. 1c). Loss of ERK activity followed a similar dose-dependence, indicating that ganetespib treatment was exerting direct effects on the MAPK pathway. Taken together, such coordinate impacts on multiple signaling cascades due to targeted HSP90 inhibition underscore the potent cytotoxic activity of ganetespib in this colon cancer cell line.

To examine whether these in vitro effects on viability and cellular signaling translated to antitumor activity in vivo, we evaluated the efficacy of single-agent ganetespib treatment on the growth of HCT 116 xenografts. The highest non-severely toxic dose of ganetespib on a weekly dosing regimen has previously been determined to be 150 mg/kg [11]. As shown in Fig. 1d, tumor volumes in mice bearing HCT 116 xenografts that were treated on this regimen were decreased by approximately half compared to those of control animals (T/C value 52 %). In addition, the schedule was well tolerated, with no toxicity or significant changes in body weight observed over the 3-week period (data not shown).

### Modulation of cell cycle protein expression by ganetespib induces growth arrest and apoptosis

**Fig. 2:**
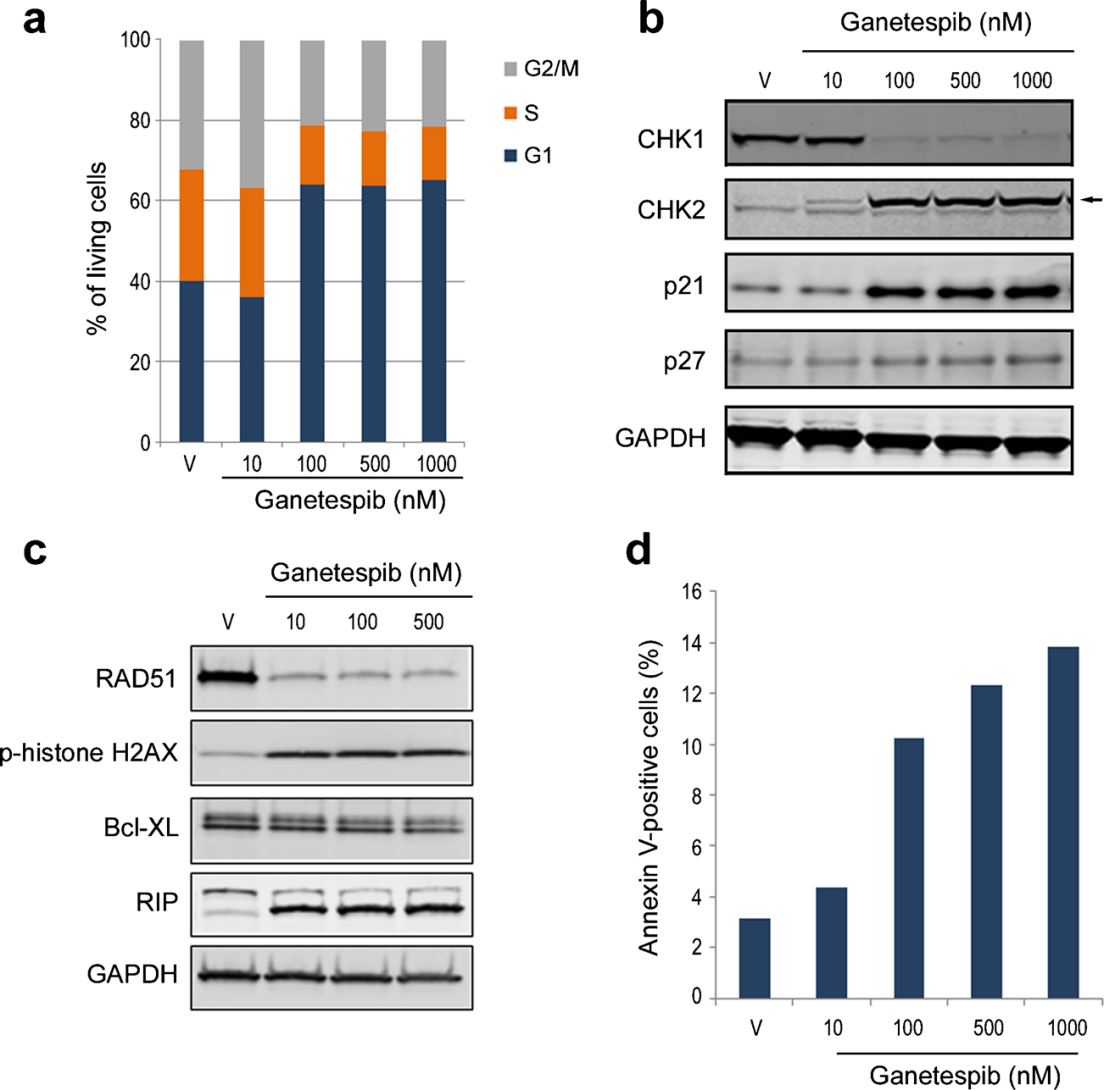
Ganetespib modulates cell cycle expression and induces growth arrest and apoptosis in HCT 116 colon cancer cells. **a** HCT 116 cells were treated with increasing concentrations of ganetespib as indicated. Cell cycle distribution was determined by flow cytometry 18 h post-treatment. **b** HCT 116 cells were treated with graded concentrations of ganetespib for 18 h. Cell lysates were immunoblotted using antibodies against CHK1, CHK2, p21 and p27. Arrow depicts higher molecular weight form of CHK2 induced following ganetespib treatment. **c** HCT 116 cells were exposed to increasing concentrations of ganetespib for 24 h. Cell lysates were immunoblotted using antibodies against RAD51, phosphorylated histone H2AX (p-histone H2AX), Bcl-XL and RIP, as indicated. **d** Quantification of apoptosis as assessed by Annexin V positivity. HCT 116 cells were exposed to increasing concentrations of ganetespib for 24 h and Annexin V/PI staining measured by flow cytometry

We have previously shown for other tumor types that, in addition to oncogenic signaling pathways, profound effects on cell cycle regulatory proteins contribute to the antitumor activity of ganetespib [13, 18]. Cell cycle analysis revealed that ganetespib exposure led to a dose-dependent G1 accumulation of HCT 116 cells, which was associated with a concomitant loss of S phase (Fig. 2a). At the molecular level, observed elevations in the levels of two protein markers expressed predominantly in the G1 phase, p21 and p27, were consistent with this cellular response (Fig. 2b). Interestingly, differential effects were seen on the expression of CHK1 and CHK2, two functionally overlapping serine/threonine kinases that play critical roles within the cell cycle as genome integrity checkpoints. While ganetespib treatment caused a potent reduction in CHK1 protein expression, this contrasted with an upregulation of CHK2, which becomes activated primarily within the context of DNA damage (Fig. 2b). In addition, activation of CHK2 requires phosphorylative events and the higher molecular weight form of the protein observed following ganetespib exposure is consistent with induced expression of the kinase in an active state (arrow). This was confirmed by loss of the higher molecular weight CHK2 following phosphatase treatment (Supplementary Fig. S1).

More extensive characterization of the impact of ganetespib on genomic integrity revealed that drug treatment of HCT 116 cells promoted degradation of the DNA damage-repair protein RAD51 and induced elevations in the phosphorylated form of histone H2AX, a sensitive indicator of DNA double-strand break formation (Fig. 2c). This was accompanied by reductions in the levels of the anti-apoptotic regulator Bcl-XL as well as a concomitant and robust increase in expression of the death domain serine/threonine kinase RIP (Fig. 2c). Taken together, this correlative loss of a pro-survival modulator with increased pro-apoptotic protein expression suggested that perturbation of the cell cycle and DNA repair pathways following ganetespib treatment ultimately triggered apoptosis. This premise was further supported by Annexin V staining data showing that HCT 116 cells treated with escalating doses of ganetespib for 24 h exhibited a dose-dependent increase in the percentage of apoptotic cells (Fig. 2d).

### Ganetespib treatment increases the radiosensitivity of HCT 116 cells in vitro

**Fig. 3:**
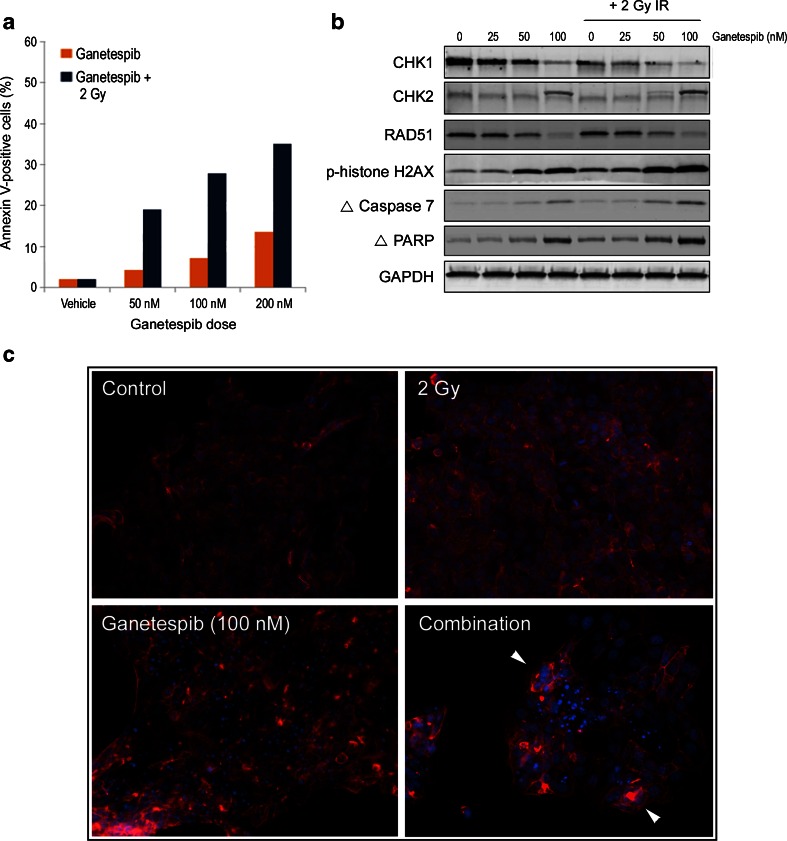
Ganetespib sensitizes HCT 116 colon cancer cells to ionizing radiation. **a** HCT 116 cells were treated with increasing doses of ganetespib either alone or in combination with 2 Gy dosing of irradiation (Ganetespib + 2 Gy). At 48 h post-IR, quantification of apoptosis was assessed by Annexin V-positivity measured by flow cytometry. **b** HCT 116 cells were treated with increasing doses of ganetespib (0, 25, 50 and 100 nM), either alone or in combination with 2 Gy irradiation. At 24 h post-treatment, lysates were prepared and immunoblotted using antibodies against CHK1, CHK2, RAD51, phosphorylated histone H2AX (p-histone H2AX), cleaved Caspase 7, and cleaved PARP. GAPDH was included as a loading control. **c** Representative images of HCT 116 cells treated with vehicle, 100 nM ganetespib, irradiated with a single dose of 2 Gy or simultaneously treated with the combination for 48 h, as indicated. Immunofluorescence was performed on cells stained for actin (red) and DAPI (blue). Arrowheads depict examples of large, multinucleated cells. Original magnification, 20×

Inhibition of HSP90 may enhance the sensitivity of tumor cells to the effects of ionizing radiation (IR). Therefore we investigated the in vitro radiosensitizing activity of ganetespib using the HCT 116 colon cancer model. Cells were treated with ganetespib either alone or in combination with a low, constant IR dose of 2 Gy. This dose level was selected since, in the absence of drug, it did not elicit any degree of apoptotic cell death at 48 h (Fig. 3a). As expected, cellular exposure to ganetespib alone resulted in a modest, dose-dependent increase in the percentages of Annexin V-positive cells by this time point. However, the degree of apoptotic induction was several fold higher in cells that underwent combined ganetespib-IR treatment (Fig. 3a). Thus, the addition of ganetespib to low dose radiation dramatically potentiated the cytotoxic effects of irradiation and to levels above those seen for drug addition alone (Fig. 3a).

Molecular changes that resulted from this cooperative enhancement of radiosensitivity were subsequently analyzed by Western blot (Fig. 3b). CHK1 is an important regulator of G2 arrest and abrogation of the G2/M checkpoint can sensitize cancer cells to IR [23]. In agreement with the data presented in Fig. 2b, CHK1 protein expression was potently destabilized following ganetespib treatment. Notably, this response was amplified in HCT 116 cells exposed to the combination of ganetespib plus IR. CHK2 activation was similarly augmented in combination treated cells, suggesting an enhanced reactive response to genotoxic stress. Consistent with this, the levels of the DNA damage repair protein RAD51 were effectively suppressed in a drug dose-dependent manner in ganetespib-IR treated cells, and overall levels of p-histone H2AX were increased by combination treatment. Taken together, these data suggest that the diminished capacity for DNA repair conferred by ganetespib may contribute to a higher degree of DNA double strand formation arising from IR exposure. As predicted by the analysis shown in Fig. 3a, levels of cleaved caspase 7 and cleaved PARP, both markers of apoptotic induction, showed higher relative expression in ganetespib-IR treated cells.

These findings were further supported by microscopic examination of HCT 116 cells (Fig. 3c). Irradiation with 2 Gy alone had no effect on nuclear morphology at 48 h, in contrast to the widespread distribution of condensed, fragmented nuclei characteristic of apoptotic cell death following 100 nM ganetespib exposure. The ganetespib-IR combination resulted in fewer viable cells at this time point, with a high percentage of those remaining showing signs of mitotic catastrophe, as evidenced by enlarged micro- and multinucleated cells, in addition to extensive nuclear condensation (Fig. 3c). Taken together, these data show that simultaneous ganetespib treatment and irradiation can induce radiosensitization and promote cell death in CRC tumor cells.

### Ganetespib potentiates the antitumor efficacy of fluoropyrimidine-based chemotherapy in CRC xenografts

**Fig. 4:**
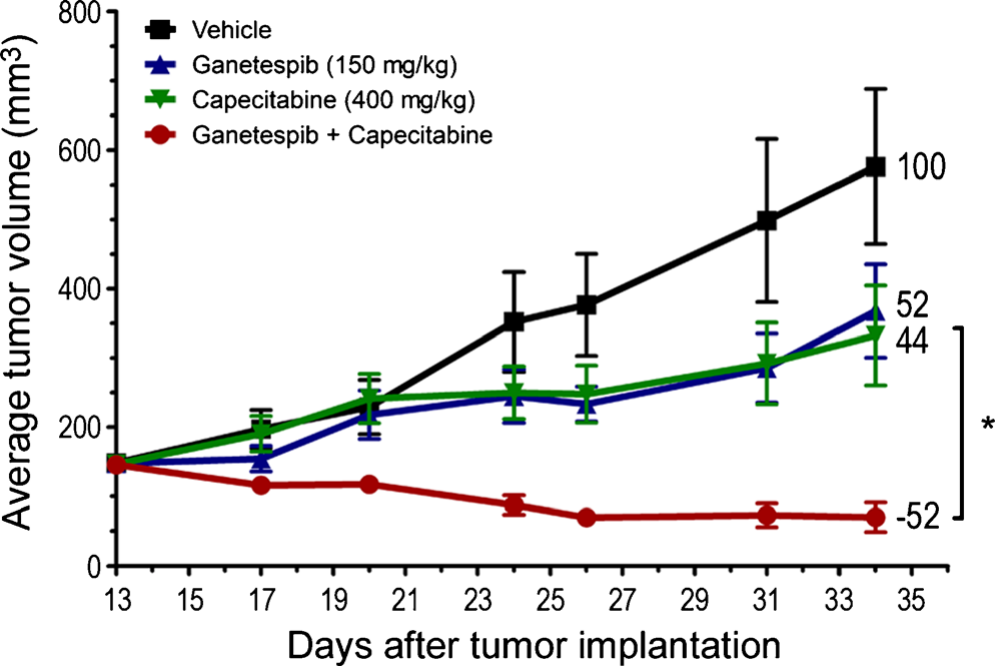
Combination ganetespib plus capecitabine treatment confers superior antitumor efficacy in CRC xenografts. Over a 3 week cycle, mice bearing established HCT 116 xenografts (*n* = 8/group) were i.v. dosed with 150 mg/kg ganetespib 1×/week or p.o dosed with 400 mg/kg capecitabine daily for 14 consecutive days, either alone or in combination. % T/C values are indicated to the right of each growth curve and the error bars are the SEM. Combination ganetespib + capecitabine treatment resulted in significant tumor regression (*, *p* < 0.05)

As presented in Fig. 1, ganetespib displayed modest single-agent efficacy in HCT 116 xenografts in vivo. Further, it has been proposed that HSP90 inhibitors, as a class, will likely be most effective in the clinical setting as part of rationally designed combination therapies [24]. In clinical practice, bolus 5-FU plus leucovorin (5-FU/LV) therapy is the standard adjuvant treatment for colon cancer. Capecitabine, an oral fluoropyrimidine and prodrug of 5-FU, has more recently become an established alternative to 5-FU/LV as first-line treatment for metastatic CRC. We therefore examined whether the addition of ganetespib to capecitabine would improve therapeutic indices in the HCT 116 xenograft model. As shown in Fig. 4, weekly administration of ganetespib (150 mg/kg) over a 3 week schedule resulted in 48 % tumor growth inhibition (T/C value, 52 %). Mice bearing HCT 116 tumors were also administered capecitabine p.o. (400 mg/kg) daily for the first 14 days on-study, and then dosing was suspended for the final 7 days (simulating the clinical dosing schedule). This regimen resulted in a similar degree of tumor growth suppression (T/C value, 44 %; Fig. 4a). Strikingly, concurrent treatment with both drugs at the same dose levels and schedule resulted in a significant improvement in antitumor activity, inducing 52 % tumor regression (*p* < 0.05 vs. capecitabine treatment alone). This finding clearly demonstrated that co-treatment with ganetespib robustly potentiated the activity of capecitabine in this model. In addition, the combination was well tolerated, with no significant loss of body weight seen after 3 weeks of dosing (data not shown).

## Discussion

The clinical management of CRC is typically dictated by the stage of the disease. Patients who present with early stage, localized tumors are amenable to curative resection surgery. Adjuvant chemotherapy is indicated for patients with Stage III disease or higher, since this modality is superior to surgical intervention alone in terms of improving disease-free and overall survival as well as preventing recurrence [25]. Chemotherapeutic regimens include the traditional cytotoxic drugs 5-FU, capecitabine, irinotecan, and oxaliplatin, with newer biologic agents bevacizumab, cetuximab, and panitumumab now being employed in combinatorial approaches for metastatic CRC [26]. Indeed, the addition of molecularly targeted agents to existing chemotherapeutic regimens has effectively doubled the survival outlook for patients with metastatic disease over the past decade [3, 26]. This approach thus serves as an encouraging paradigm for the design of novel strategies to provide continued improvements for patients with advanced CRC.

Here we investigated the antitumor activity profile of ganetespib, a second-generation inhibitor of HSP90 currently under clinical evaluation in multiple human trials. Ganetespib potently reduced cell viability in vitro in a panel of CRC cell lines, with drug exposure resulting in the dose-dependent destabilization of multiple HSP90 client proteins, including MET, EGFR, IGF-1R and AKT. Overall, the combinatorial blockade of multiple key signaling components required for CRC cell growth and survival, perturbation of cell cycle regulation, and subsequent induction of apoptosis were sufficient to account for the potent cytotoxic activity of this agent. These findings complement and extend those of a recent study showing that ganetespib exerts robust antiangiogenic activity in preclinical models of CRC, through disruption of HIF-1α and STAT3 signaling [27].

Despite their therapeutic promise, no HSP90 inhibitors are currently approved for cancer treatment. The most impressive clinical effects observed with HSP90 inhibitor monotherapy have arisen in defined subsets of tumors that exhibit a high degree of oncogenic dependence on particular client proteins e.g., ALK-driven non-small cell lung cancer and HER2-amplified breast cancer [24]. The proteins that show the highest frequency of alteration as part of colorectal tumorigenesis, APC and KRAS, are themselves not HSP90 clients. Thus, in general, this malignancy might not be expected to be highly responsive to targeted HSP90 therapeutic intervention. A possible exception, however, involves the serine/threonine kinase BRAF, mutant forms of which are highly reliant on the chaperone function of HSP90 for stability and function [28]. Mutations in BRAF account for only approximately 5–15 % of CRC cases [29], although their presence has been strongly associated with poorer outcomes [30, 31]. From our screen of 15 human CRC lines it was found that the two most sensitive to ganetespib exposure (RKO and LS-411 N) harbor activating BRAF^V600E^ mutations. We have recently reported similar potent activity for ganetespib in melanoma lines that show acute dependence on mutant BRAF^V600E^ [22]. For the CRC patient who achieved a partial response in the initial Phase I evaluation of ganetespib, no tumor tissue was available for mutational analysis [21], and thus the underlying molecular phenotype that dictated a high sensitivity to single-agent ganetespib therapy could not be determined. In a subsequent Phase II study of ganetespib in heavily pre-treated metastatic colorectal patients [NCT01111838] no objective responses were observed, although 2/15 evaluable patients (both with G12V-mutant KRAS tumors) achieved durable stable disease [32]. In that trial, all tumors were wild type for BRAF expression. Overall, while it remains to be determined whether mutated BRAF represents an actionable target for HSP90 inhibitor therapy in CRC, our findings suggest that the full potential of ganetespib is likely to be realized as part of novel combinatorial approaches in this disease.

As part of the characterization of the prototypical ansamycin family of HSP90 inhibitors, including geldanamycin and its derivatives 17-allylamino-17-demethoxygeldanamycin (17-AAG), and 17-dimethylaminoethylamino-17-demethoxygeldanamycin (17-DMAG), it was discovered that these compounds could sensitize a variety of tumor cell lines to the cytotoxic effects of IR [33–36]. Similar activity has been seen with second-generation HSP90 inhibitors [37–39], suggesting that targeted HSP90 blockade represents a conserved and potentially attractive strategy for enhancing cancer cell radiosensitivity. Consistent with such reports, ganetespib acted as a radiosensitizer to potentiate the effects of low dose IR in the HCT 116 model. At the molecular level, it was found that combined HSP90 inhibition and radiation impacted several overlapping pathways that led to cell cycle dysregulation, diminished DNA repair capacity and enhanced apoptosis. In response to DNA damage, cells typically activate ataxia telangiectasia-mutated (ATM)-CHK2 and/or ATM and Rad3 related (ATR)-CHK1 signaling pathways to arrest the cell cycle and initiate DNA repair [40]. CHK1 plays an important role in the activation and maintenance of the G2/M checkpoint and, importantly, is an established HSP90 client. Accordingly, ganetespib exposure alone was sufficient to destabilize CHK1 expression in HCT 116 cells and this effect was augmented when cells were exposed to combined ganetespib-IR treatment. These data suggested that the integrity of any radiation-induced G2/M checkpoint was markedly compromised with concurrent ganetespib treatment. CHK2 becomes activated in response to double-strand DNA breaks (including those arising as a direct result of IR) and propagates such damage signals through downstream targets involved in cell cycle progression and apoptosis [40]. In this regard, analysis of CHK2 regulation revealed that the induction of activated CHK2 protein levels was potentiated by radiation exposure, even using a largely ineffective dose (2 Gy)—reflecting the higher degree of DNA double-strand breakage resulting from combination treatment. This was linked to an impaired DNA repair capability due to the ganetespib-driven loss of RAD51, a critical protein for the homologous recombination pathway of DNA double-strand break repair. Indeed, when HCT 116 cells were co-treated with ganetespib, damage repair mechanisms appeared to be inhibited following irradiation as evidenced by robust increases in phosphorylated histone H2AX expression. It is reasonable to suggest therefore that the genotoxic stress induced by the interplay of these cellular responses was sufficient to account for the increased cell death observed when the two modalities of HSP90 blockade and irradiation were combined.

An important finding of this study, and one with clear clinical relevance, was the capacity of ganetespib to significantly improve the efficacy of fluoropyrimidine therapy in HCT 116 xenografts. For five decades now, 5-FU has played an indispensable role in CRC treatment, both in the curative and palliative settings [3]. 5-FU acts as an antimetabolite to disrupt DNA and RNA synthesis and repair, ultimately leading to cancer cell death. It is the backbone of the FOLFOX (5-FU/LV plus oxaliplatin) and FOLFIRI (5-FU/LV plus irinotecan) combination therapies that represent the standard first line cytotoxic regimens for metastatic CRC patients. The two regimens are equivalent in terms of efficacy, with selection of one over the other largely dependent on different toxicity profiles and patient performance status [41]. Due to a short half-life and significant variations in bioavailability, 5-FU requires intravenous infusion (bolus and/or continuous) and the first oral prodrug formulation, capecitabine, received FDA approval in 2005 for adjuvant monotherapy use [42]. HCT 116 cells exhibited a high tolerance to 5-FU treatment in vitro, believed to be linked to their DNA mismatch-repair deficient phenotype [43]. In vivo, capecitabine administered on a clinically relevant dosing schedule suppressed HCT 116 xenograft growth by over half. Single-agent ganetespib treatment showed a similar, modest degree of tumor growth inhibition in xenografted HCT 116 tumors, comparable to what has previously been reported for 17-DMAG [44]. However, when the two drugs were combined, we observed a significant improvement in antitumor efficacy resulting in greater than 50 % tumor regression. A synergistic interaction between 5-FU and the HSP90 inhibitor NVP-AUY922 has previously been reported in bladder cancer cell lines; no correlative translation into in vivo efficacy was shown in that study [45]. Thus, to our knowledge, this is the first demonstration of combinatorial benefit between a small molecule HSP90 inhibitor and the fluoropyrimidine in CRC-derived tumors. Further, our data showed that not only did the addition of ganetespib to 5-FU-based therapy potentiate the activity of the cytotoxic agent, but that targeted HSP90 inhibition could overcome the intrinsic 5-FU resistant phenotype of HCT 116 cells.

The most dramatic recent clinical advances for patients with metastatic CRC have resulted from the integration of molecularly targeted agents, such as the anti-VEGF antibody bevacizumab and the EGFR antagonists cetuximab and panitumumab, into existing therapies [26]. An important lesson gleaned from the development of those new therapeutic strategies was that each of the biologics displayed minimal clinical activity as single agents and their full benefit was only realized when they were combined with standard treatment regimens [3]. It is reasonable to suggest that this profile is likely to be similar for the future application of selective HSP90 inhibitors in CRC. Importantly, just as KRAS mutations emerged as negative molecular predictors of response to EGFR-targeted agents in this disease [46], the identification of predictive biomarkers that identify those individuals most likely to receive therapeutic benefit from HSP90 blockade remains an ongoing clinical consideration. The findings presented here suggest a compelling rationale for exploiting the chemosensitizing activity and capacity to overcome fluoropyrimidine resistance displayed by ganetespib. In light of these considerations, further investigation into the potential benefits of ganetespib as an adjunct to 5-FU-based therapy is warranted and a Phase I trial evaluating ganetespib in combination with capecitabine and radiation in rectal cancer [NCT01554969] has recently been initiated.

In summary, we have shown that ganetespib exhibits robust cytotoxic activity in preclinical models of CRC due to coordinate effects on multiple cellular signaling pathways, DNA repair mechanisms, and cell cycle progression. The data presented here suggest that ganetespib may offer considerable promise for therapeutic intervention in CRC, particularly as part of novel combinatorial strategies with existing standard of care regimens. Moreover these findings establish a novel framework for the design of future ganetespib-based approaches to improve patient outcomes in this disease.

## Electronic supplementary material

Below is the link to the electronic supplementary material.

Fig. S1 (GIF 37 kb)

High Resolution Image (TIFF 120 kb)
