# Supplementary figures and images for: The HSP90 inhibitor ganetespib has chemosensitizer and radiosensitizer activity in colorectal cancer
Source: Invest New Drugs. 2014 Apr 1;32(4):577–86. doi: 10.1007/s10637-014-0095-4 (PMC4101249; doi:10.1007/s10637-014-0095-4)

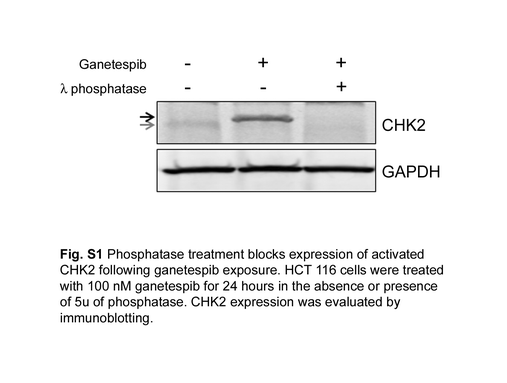

Supplement: Supplementary file 1 — (GIF 37 kb) [file 10637_2014_95_Fig5_ESM.gif]

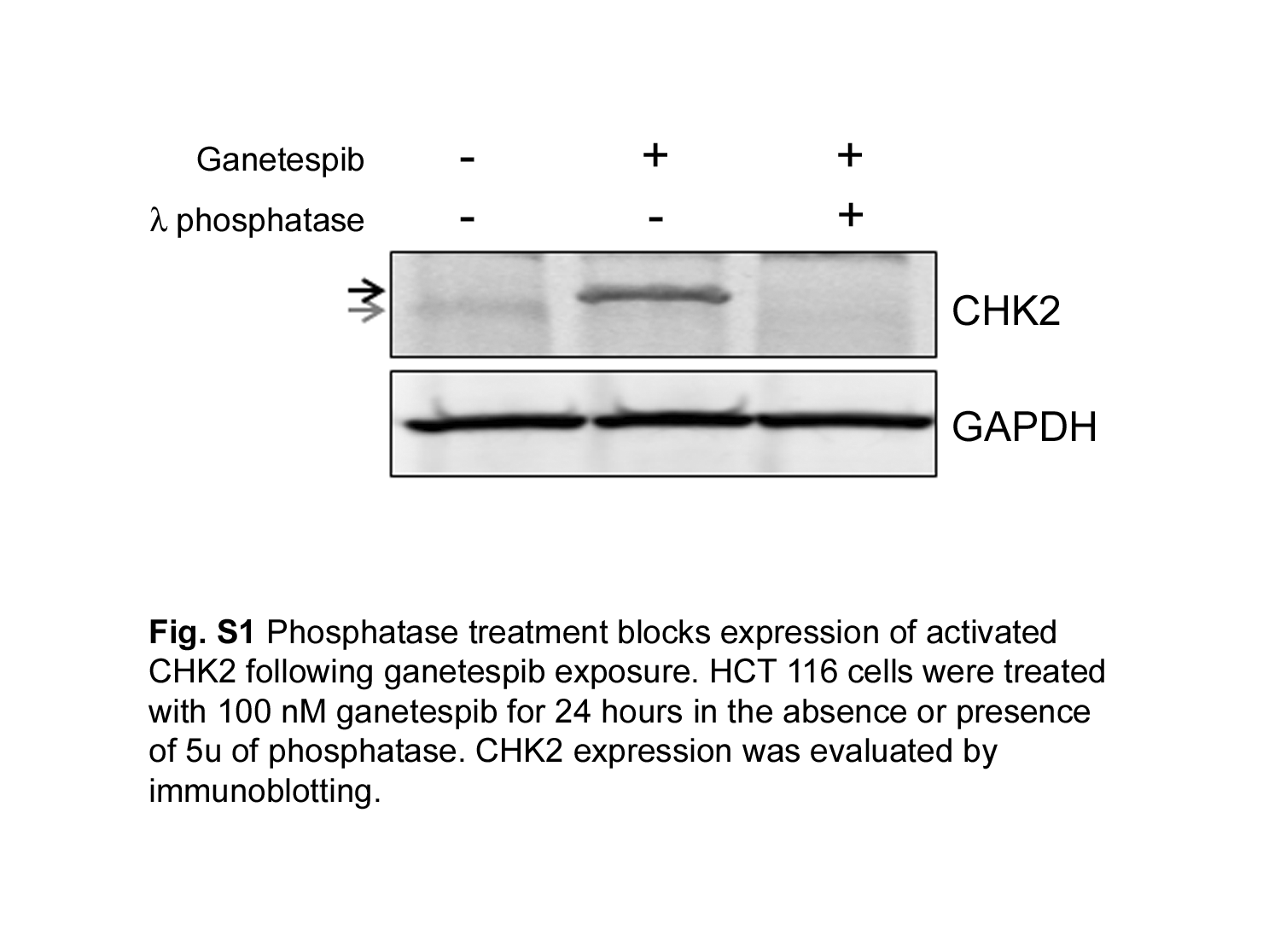

Supplement: Supplementary file 2 — High Resolution Image (TIFF 120 kb) [file 10637_2014_95_MOESM1_ESM.tif]
